# Dataset characterizing tailoring techniques, challenges, impacts and metrics in Large-Scale agile development

**DOI:** 10.1016/j.dib.2026.113360

**Published:** 2026-10-19

**Authors:** F. Gracia-Ahufinger, JAVIER J. Gutiérrez, J.A. García-García, N. Sánchez-Gómez, M.J. Escalona-Cuaresma

**Affiliations:** Computer Languages and Systems Department, Computer Engineering School, University of Seville, Seville, Andalucia, Spain

**Keywords:** Large-scale agile development, Agile tailoring, Agile techniques, Agile challenges, Agile impacts, Agile metrics, Agile dataset, Systematic mapping study

## Abstract

Large-Scale Agile Development refers to the coordination of Agile practices across multiple teams, departments and globally distributed sites. While Agile is the software development industry standard, Large-Scale Enterprises frequently struggle with one-size-fits-all adoption and require specific tailoring to fit their complex organizational structures. Consequently, Agile Techniques Tailoring, the adaptation of specific frameworks like Scrum, Kanban or eXtreme Programming to fit complex structures and organizational culture, has become critical for Large-Scale Enterprises seeking to improve flexibility and time-to-market. This tailoring approach has been extended over time to scaled frameworks such as Scaled Agile Framework, Disciplined Agile Delivery, Large-Scale Scrum or the Spotify Model. This dataset provides a comprehensive characterization of evidence generated through a Systematic Mapping Study which covers publications between 2015 and 2025, following a rigorous five-phase screening process based on inclusion and exclusion criteria. The dataset includes a Characterization Scheme containing 36 attributes and a full inventory of the primary study list used for data extraction, including challenges, positive/negative impact techniques, the specific metrics used to evaluate success and the approaches used to deliver value. The dataset allows researchers and practitioners to navigate empirical evidence regarding Agile transformation, identify research gaps and compare the effectiveness of different Agile techniques.

Specifications Table

**Table:** 

Subject	Computer Sciences
Specific subject area	Characterization of tailoring techniques, challenges, impacts and metrics in Large-Scale Agile Development
Type of data	CSV (comma “,” delimiter), JSON (generated with Visual Studio Code), XLSX Analyzed and Processed
Data collection	Data was extracted through a Systematic Mapping Study [1] of empirical literature between 2015 and 2025 across five major digital libraries. A rigorous five-phase screening protocol was applied to initial search results to identify relevant research based on predefined inclusion/exclusion criteria. Data extraction used Large Language Models as coding assistant for pattern recognition and structured thematic synthesis. This process was governed by an author-in-the-loop protocol, where researchers manually validated all extracted data points against a 36-attribute Characterization Scheme to ensure high reliability and traceability.
Data source location	https://zenodo.org
Data accessibility	Repository name: Zenodo Data identification number: 10.5281/zenodo.19944192 Direct URL to data: https://doi.org/10.5281/zenodo.19944192 Instructions for accessing these data: The data is openly accessible under a Creative Commons Attribution 4.0 International license.
Related research article	None

## 1 Value of the Data

• This dataset provides a structured inventory offering comprehensive evidence for the rapidly evolving field of Large-Scale Agile Development. It explicitly characterizes the quantitative gap in Agile Scaling research by categorizing specific metrics used in the literature to validate transformation success.
• Through a detailed 36-attribute Characterization Scheme, the dataset enables full traceability between high-level organizational challenges and specific empirical evidence, allowing practitioners to benchmark their own transformation models against established industry trends.
• The dataset provides a centralized repository enabling researchers to quickly access a decade of empirical evidence without repeating the search process and utilize this data to perform secondary analyzes on specific subsets.
• Practitioners can use the data to identify proven tailoring techniques and anticipate common challenges, such as inter-team coordination and cultural resistance, before beginning a transformation.
• The data highlights the quantitative gap in the field, showing that most success claims are based on qualitative perceptions rather than hard metrics.

The landscape of publicly available Agile datasets remain an area for exploration; indeed, a targeted research for Agile-focused datasets within this journal, Data in Brief, identified only a single direct precedent: the multimodal experimental dataset in [2]. The referred work captures deep verbal and non-verbal interactions, including facial expressions, gaze and mimicry, within simulated Agile teams during specific tasks like Planning Poker. While this resource is valuable for predicting project success or understanding team-level dynamics, they do not address the specific complexities of Large-Scale Agile Development. Our dataset is more relevant because it specifically bridges this gap by characterizing the tailoring techniques, challenges and impacts inherent in those environments. Unlike existing datasets that measure general task metrics or project-wide outcomes, our work provides a structured, multi-dimensional view of how Agile is adapted at scale, offering a unique resource for researchers focused on organizational-level transformations and the methodological adjustments required for large development programs.

Additionally, foundational studies such as the systematic review by Dikert et al. [3] provide essential taxonomies of challenges and success factors across industrial cases, while mapping studies by Dingsøyr et al. [4] have effectively chartered the temporal growth and general research interest of the domain. However, this dataset explicitly characterizes the quantitative gap by identifying specific metrics used or missing in the literature to validate transformation outcomes, unlike prior reviews that rely heavily on qualitative summaries of success factors. Finally, this dataset offers a unique operational tool for practitioners to benchmark their models against established industry trends, a level of utility not presented in purely descriptive literature syntheses.

## 2 Background

The compilation of this dataset was motivated by the evolving landscape of Large-Scale Agile Development and the lack of structured empirical evidence regarding how Large-Scale Enterprises tailor Agile frameworks to their specific contexts. While Agile frameworks were originally designed for small, co-located teams, Large-Scale Enterprises face significant friction when scaling these techniques across hundreds of developers and globally distributed sites. Literature frequently reports high failure rates in these transformations, often attributed to cultural friction, coordination chaos [5,6] and the Water-Scrum-Fall phenomenon [7].

A primary methodological driver for generating this data was the identified quantitative gap: the significant disparity between industry claims of Agile success and the availability of rigorous empirical evidence to validate those claims. This dataset was compiled through a Systematic Mapping Study to systematically structure the research area and identify prevalent challenges, impacts and metrics. By providing a structured and traceable inventory of the primary studies analyzed, this dataset adds value through its granular characterization attributes.

## 3 Data Description

The dataset is structured around a comprehensive Characterization Scheme defined in Table 1, detailing 36 attributes organized into several logical dimensions. The final dataset consists of 113 individual records, with each row representing one of the primary studies identified through the five-phase screening protocol.

• Study Metadata: Fields for identification and traceability, including ID, Title, Authors, Keywords and Year of publication, Forum and the DOI reference.
• Foundational Agile Concepts: Attributes that evaluate the study’s alignment with core Agile values, such as Methodology, classifying Agile as a methodology, Manifesto [8], adherence to theory or practice, and the presence of Socio-Technical Factors.
• Methodological Rigor: Technical metadata including Publication Type, Research Type, Research Method, Research Method Group, Research Method Design, Research Method Design Motivation, Validation Method and Validation Method Group employed.
• Enterprise Context: Categorization of the research environment, specifying the Research Industry, Research Company Names and the research Company Size.
• Agile Specifics: Identification of the Main Frameworks Analyzed, the specific Scaling Approach used, and any Other Frameworks mentioned or compared.
• Thematic Evidence Extraction: The core data points related to the research questions, capturing Challenges, Positive and Negative Impact Techniques, Positive and Negative Impact Definitions, Positive and Negative Impact Metrics and Value Delivery Approaches.
• Synthesized Findings: Qualitative summaries providing a Focus, Short Description, Key Findings and proposed Future Research Lines.

**Table 1:** Characterization Scheme.

Attribute	Description	Type	Overall field completeness
ID	Unique identifier assigned to each study	String	100 %
Title	Full name of the research study	String	100 %
Authors	Researchers who conducted and published the study	String separated by |	100 %
Keywords	Terms or phrases that identify the core topics of the study	String separated by |	98.2 %
Year	Year when the study was published	4-digit number	100 %
Forum	Specific journal, conference or workshop where the study appeared	String	100 %
DOI	Unique, persistent alphanumeric string assigned to provide a permanent, reliable link to the work	String	100 %
Methodology	Indicates whether the study classifies Agile frameworks as methodologies	Binary: 1 (Yes), 0 (No)	100 %
Manifesto	Indicates if the study explicitly aligns with or evaluates adherence to the Agile Manifesto principles	Enumeration: T (Theory), P (Practice), T/P, N (No)	100 %
Publication Type	Category of the publication, aligned with the document-type labels of the source digital libraries	Enumeration: Conference Paper, Journal Article, Research Paper, Technical Paper, Workshop Paper	100 %
Challenges	Challenges found when applying Agile at scale	String	91.2 %
Positive Impact Techniques	Techniques that lead to positive outcomes in Agile implementations	String	61.1 %
Positive Impact Definitions	Definitions of what constitutes a positive impact in Agile implementations	String	61.1 %
Positive Impact Metrics	Metrics used to measure positive impacts in Agile implementations	String	61.1 %
Negative Impact Techniques	Techniques that lead to negative outcomes in Agile implementations	String	76.1 %
Negative Impact Definitions	Definitions of what constitutes a negative impact in Agile implementations	String	76.1 %
Negative Impact Metrics	Metrics used to measure negative impacts in Agile implementations	String	63.7 %
Value Delivery Approaches	Approaches to delivering value in Agile implementations	String	98.2 %
Socio-Technical Factors	Analysis of how the study addresses the interplay between social and technical systems	String	89.4 %
Research Type	Category of research performed	Enumeration: Evaluation Research, Experience Paper, Solution Proposal, Validation Research, Philosophical Paper	100 %
Research Method	Specific scientific design applied	String	100 %
Research Method Group	Broader grouping of the research method	Enumeration: Action Research, Case Study, Comparative Analysis, Experiment, Grounded Theory, Review, Survey, Other	100 %
Research Method Design	Whether the study relies on numerical data, descriptive data or a combination	Enumeration: Quantitative, Qualitative, Mixed Methods	100 %
Research Method Design Motivation	Rationale provided for selecting the specific data type and analysis	String	100 %
Validation Method	Specific technique used to verify findings	String	100 %
Validation Method Group	Broader categorization of the validation approach	String	54.9 %
Research Industry	Business sector where the research was conducted	String separated by |	100 %
Research Company Names	Specific names or pseudonyms of the organizations involved in the study	String separated by |	87.6 %
Research Company Size	Classification of the organizations based on their scale	String separated by |	90.3 %
Main Framework analyzed	Primary Agile framework investigated	String separated by |	92.9 %
Scaling Approach	Specific tactic or model used to scale Agile techniques	String separated by |	80.5 %
Other Frameworks	Comprehensive list of all scaling frameworks mentioned or compared in the study	String separated by |	100 %
Focus	Core thematic area or subject the study concentrates on	String	100 %
Short Description	Brief overview of the study’s scope and implementation	String	100 %
Key Findings	Primary conclusions, results or lessons learned from the study	String	100 %
Future Research Lines	Identified areas or questions that require further investigation	String	91.2 %

*Publication Type* attribute categorizes the formal dissemination medium of each primary study. To maintain alignment with indexing used in digital libraries, specifically Web of Science (WoS), this field retains values such as *Research Paper* and *Technical Paper* alongside traditional venue categories, *Journal Article, Conference Paper* and *Workshop Paper*. This choice was made during the initial design of the Characterization Scheme to mirror WoS document type metadata directly, avoiding confusion with specific conference or workshop venue names. Categorical and methodological attributes of the research design, like *Experience Paper, Evaluation Research* or *Solution Proposal*, are independently captured under the *Research Type* field to prevent conceptual overlap.

## 4 Illustrative Data Examples

The tangible reuse value of this dataset is best demonstrated through its capacity for targeted evidence-based querying with an example use case. A researcher or practitioner facing “*Inter-team Coordination and Dependency Management*”, the most frequently reported challenge in the dataset, can utilize the following querying workflow (Fig. 1):

• Isolate relevant records: By filtering the “*Challenges*” column for this specific string, a user can instantly isolate a targeted subset of records.
• Cross-reference strategic evidence: Within this filtered subset, the user can cross-reference the “*Positive impact techniques*” column to identify empirically validated strategies. For instance, the data identifies techniques like “*Continuous Deployment*” or “*Product Owner Synchronization*” as specific remedies for coordination bottlenecks.
• Identify validation contexts: To understand exactly how success was measured in real-world industrial contexts, the user can consult the “*Positive impact metrics*” column. This identifies specific benchmarks used in the literature, such as “*Deployment Frequency*” or “*OKR Achievement*”, providing the necessary hard data to support transformation decisions.

**Fig. 1:**
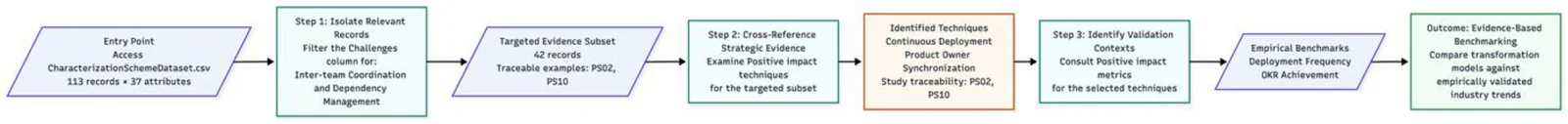
Querying workflow.

To demonstrate the multidimensional traceability and the richness of the Characterization Scheme, Table 2 provides a representative data snippet focused on the most prevalent challenges in the primary studies for the mentioned use case. This sample illustrates how the Characterization Scheme Dataset connects specific organizational hurdles to empirically validated techniques and the metrics used to measure their success.

**Table 2:** Representative data snippet for example use case.

Study ID	Challenges	Positive Impact Techniques	Positive Impact Metrics
PS02	Coordination breakdowns and dependencies that undermine team autonomy and cause communication overload.	Continuous deployment, team autonomy, and second-generation coordination mechanisms.	Capability to deliver on time, within budget, and with expected quality.
PS06	Balancing team autonomy with organizational alignment and planning misalignment between team and inter-team levels.	Objectives and Key Results (OKR) framework, Communities of Practice (CoPs), and Inter-team Stand-ups.	OKR Achievement and TOPS framework assessment.
PS10	Cultural obstacles, difficulties coordinating multiple teams working on a single product, and risks of low motivation.	Continuous Deployment/Delivery, Continuous Integration, and Management 3.0 principles.	Automation Percentage and Stakeholder and Employee Satisfaction.
PS63	Tensions with traditional management regarding goal-settings, accuracy expectations, and “deadline thinking”.	Hybrid Approaches, framework tailoring, and Shared Mental Models and Trust Building.	Business Value Metric or OKR Achievement and Psychological Safety.

## 5 Experimental Design, Materials and Methods

The primary method for data acquisition was a Systematic Mapping Study [1] designed to structure the research area of tailoring Agile techniques at scale. The scope of the study was to answer the following Research Questions:

1. What challenges are most frequently reported during the scaling and tailoring of Agile techniques in Large-Scale Enterprises?
2. What tailoring techniques are described in the literature and how are their impacts defined?
3. What outcomes and metrics are used to evaluate the impact of these techniques?
4. What approaches are adopted to promote value delivery in Large-Scale Enterprises?

The research strategy was constructed by identifying key concepts through an initial search string run in Google Scholar to define representative keywords (Table 3).

**Table 3:** Keywords used to build search string.

Concept	Keywords
Theme	agile, scrum, kanban, extreme programming, xp
Adaptation	tailor, adapt, custom, scale
Context	large-scale, large-scale enterprise, large-scale corporation, large-scale company, large-scale project
Tools	technique, practice, method, adopt, implement
Influence	impact, value, measure, inspect, evaluate, assess

The search involved five major digital libraries: IEEE Xplore, Web of Science, Springer Nature Link, Scopus and ACM Digital Library. For each of them a Search Query was run in order to retrieve a first set of candidates articles (Table 4).

**Table 4:** Digital Libraries Search Strings.

Digital Library	Query
IEEE Xplore	("Abstract":"agile" OR "Abstract":"scrum" OR "Abstract":"kanban" OR "Abstract":"extreme programming" OR "Abstract":"xp") AND ("Abstract":"tailor*" OR "Abstract":"adapt*" OR "Abstract":"custom*" OR "Abstract":"scal*") AND ("Abstract":"large-scale" OR "Abstract":"large-scale enterprise" OR "Abstract":"large-scale corporation" OR "Abstract":"large-scale compan*" OR "Abstract":"large-scale project") AND ("Abstract":"technique" OR "Abstract":"practice" OR "Abstract":"method" OR "Abstract":"adopt*" OR "Abstract":"implement*" OR "Abstract":"impact" OR "Abstract":"value" OR "Abstract":"measure" OR "Abstract":"inspect*" OR "Abstract":"evaluat*" OR "Abstract":"assess*")
Web of Science	AB=(("agile" OR "scrum" OR "kanban" OR "extreme programming" OR "xp") AND ("tailor*" OR "adapt*" OR "custom*" OR "scal*") AND ("large-scale" OR "large-scale enterprise" OR "large-scale corporation" OR "large-scale compan*" OR "large-scale project") AND ("technique" OR "practice" OR "method" OR "adopt*" OR "implement*" OR "impact" OR "value" OR "measure" OR "inspect*" OR "evaluat*" OR "assess*")) OR TI=(("agile" OR "scrum" OR "kanban" OR "extreme programming" OR "xp") AND ("tailor*" OR "adapt*" OR "custom*" OR "scal*") AND ("large-scale" OR "large-scale enterprise" OR "large-scale corporation" OR "large-scale compan*" OR "large-scale project") AND ("technique" OR "practice" OR "method" OR "adopt*" OR "implement*" OR "impact" OR "value" OR "measure" OR "inspect*" OR "evaluat*" OR "assess*")) OR AK=(("agile" OR "scrum" OR "kanban" OR "extreme programming" OR "xp") AND ("tailor*" OR "adapt*" OR "custom*" OR "scal*") AND ("large-scale" OR "large-scale enterprise" OR "large-scale corporation" OR "large-scale compan*" OR "large-scale project") AND ("technique" OR "practice" OR "method" OR "adopt*" OR "implement*" OR "impact" OR "value" OR "measure" OR "inspect*" OR "evaluat*" OR "assess*"))
Springer Nature Link	("agile" OR "scrum" OR "kanban" OR "extreme programming" OR "xp") AND ("tailor*" OR "adapt*" OR "custom*" OR "scal*") AND ("large-scale" OR "large-scale enterprise" OR "large-scale corporation" OR "large-scale compan*" OR "large-scale project") AND ("technique" OR "practice" OR "method" OR "adopt*" OR "implement*" OR "impact" OR "value" OR "measure" OR "inspect*" OR "evaluat*" OR "assess*")
Scopus	TITLE-ABS-KEY (("agile" OR "scrum" OR "kanban" OR "extreme programming" OR "xp") AND ("tailor*" OR "adapt*" OR "custom*" OR "scal*") AND ("large-scale" OR "large-scale enterprise" OR "large-scale corporation" OR "large-scale compan*" OR "large-scale project") AND ("technique" OR "practice" OR "method" OR "adopt*" OR "implement*" OR "impact" OR "value" OR "measure" OR "inspect*" OR "evaluat*" OR "assess*"))
ACM Digital Library	[[Title: "agile"] OR [Title: "scrum"] OR [Title: "kanban"] OR [Title: "extreme programming"] OR [Title: "xp"]] AND [[Title: "tailor*"] OR [Title: "adapt*"] OR [Title: "custom*"] OR [Title: "scal*"]] AND [[Title: "large-scale"] OR [Title: "large-scale enterprise"] OR [Title: "large-scale corporation"] OR [Title: "large-scale compan*"] OR [Title: "large-scale project"]] AND [[Title: "technique"] OR [Title: "practice"] OR [Title: "method"] OR [Title: "adopt*"] OR [Title: "implement*"] OR [Title: "impact"] OR [Title: "value"] OR [Title: "measure"] OR [Title: "inspect*"] OR [Title: "evaluat*"] OR [Title: "assess*"]] OR [[Abstract: "agile"] OR [Abstract: "scrum"] OR [Abstract: "kanban"] OR [Abstract: "extreme programming"] OR [Abstract: "xp"]] AND [[Abstract: "tailor*"] OR [Abstract: "adapt*"] OR [Abstract: "custom*"] OR [Abstract: "scal*"]] AND [[Abstract: "large-scale"] OR [Abstract: "large-scale enterprise"] OR [Abstract: "large-scale corporation"] OR [Abstract: "large-scale compan*"] OR [Abstract: "large-scale project"]] AND [[Abstract: "technique"] OR [Abstract: "practice"] OR [Abstract: "method"] OR [Abstract: "adopt*"] OR [Abstract: "implement*"] OR [Abstract: "impact"] OR [Abstract: "value"] OR [Abstract: "measure"] OR [Abstract: "inspect*"] OR [Abstract: "evaluat*"] OR [Abstract: "assess*"]] OR [[Keywords: "agile"] OR [Keywords: "scrum"] OR [Keywords: "kanban"] OR [Keywords: "extreme programming"] OR [Keywords: "xp"]] AND [[Keywords: "tailor*"] OR [Keywords: "adapt*"] OR [Keywords: "custom*"] OR [Keywords: "scal*"]] AND [[Keywords: "large-scale"] OR [Keywords: "large-scale enterprise"] OR [Keywords: "large-scale corporation"] OR [Keywords: "large-scale compan*"] OR [Keywords: "large-scale project"]] AND [[Keywords: "technique"] OR [Keywords: "practice"] OR [Keywords: "method"] OR [Keywords: "adopt*"] OR [Keywords: "implement*"] OR [Keywords: "impact"] OR [Keywords: "value"] OR [Keywords: "measure"] OR [Keywords: "inspect*"] OR [Keywords: "evaluat*"] OR [Keywords: "assess*"]]

Specific inclusion and exclusion criteria were applied to each digital library query, as follows:

• For every digital library, only articles published from 2015 onwards were considered. Between 2010 and 2015 Scrum’s first guide, Scaled Agile Framework (SAFe), Large-Scale Scrum (LeSS), Disciplined Agile Delivery (DAD) and the Spotify Model were released and adopted in the industry. This is why 2015 was chosen as the cut-off year for the inclusion and exclusion criteria, focusing the research on the last decade. Additionally, only works published in English were included.
• For IEEE Xplore only Conference and Journal publications were included.
• For Web of Science the following criteria were considered to include articles of the search, Document Types: Article and Proceeding Paper; Web of Science Categories: Computer Science Software Engineering, Computer Science Theory Methods, Computer Science Information Systems and Management; Research Areas: Computer Science, Business Economics and Engineering.
• For Springer Nature Link the following criteria were considered to include articles of the search, Subjects: Enterprise Architecture, Method Development and Software Engineering; Disciplines: Computer Science, Engineering and Business and Management; Subdisciplines: Operating systems and Software engineering / programming, Software engineering, Management of computing and information systems, Information systems applications (incl. Internet) and It in business.
• For Scopus the following criteria were considered to include articles of the search, Source type: Journal and Conference proceeding; Subject area: Computer science, Engineering and Business, Management and Accounting.
• For ACM Digital Library, Research Articles and Short Papers were included.

The selection of primary studies followed a rigorous five-phase screening protocol to ensure transparency and reproducibility (Fig. 2):

• Phase 1 (P1 – Identification): An automated search was executed across the selected libraries using specific Boolean queries in Table 4, retrieving an initial set of 1415 candidate articles. Additionally, a manual review of XP and PROFES conference proceeding since 2015 was performed, yielding 47 additional articles for a total of 1462 initial candidate articles.
• Phase 2 (P2 – Screening Duplicates): Overlapping indexing was resolved by removing duplicated records, leaving 1019 unique publications.
• Phase 3 (P3 – Title Screening): A preliminary relevance assessment was performed by reading the titles, which reduced the set to 313 papers.
• Phase 4 (P4 – Secondary Studies): Specific exclusion criteria were applied to remove secondary studies such as Systematic Mapping or Systematic Literature Review, purely theoretical discussions, and questionnaires lacking direct industrial application, resulting in 256 papers.
• Phase 5 (P5 – Abstract Screening): A detailed single-reviewer assessment of each abstract was conducted to ensure alignment with the research questions, resulting in the final selection of 113 primary studies.

**Fig. 2:**
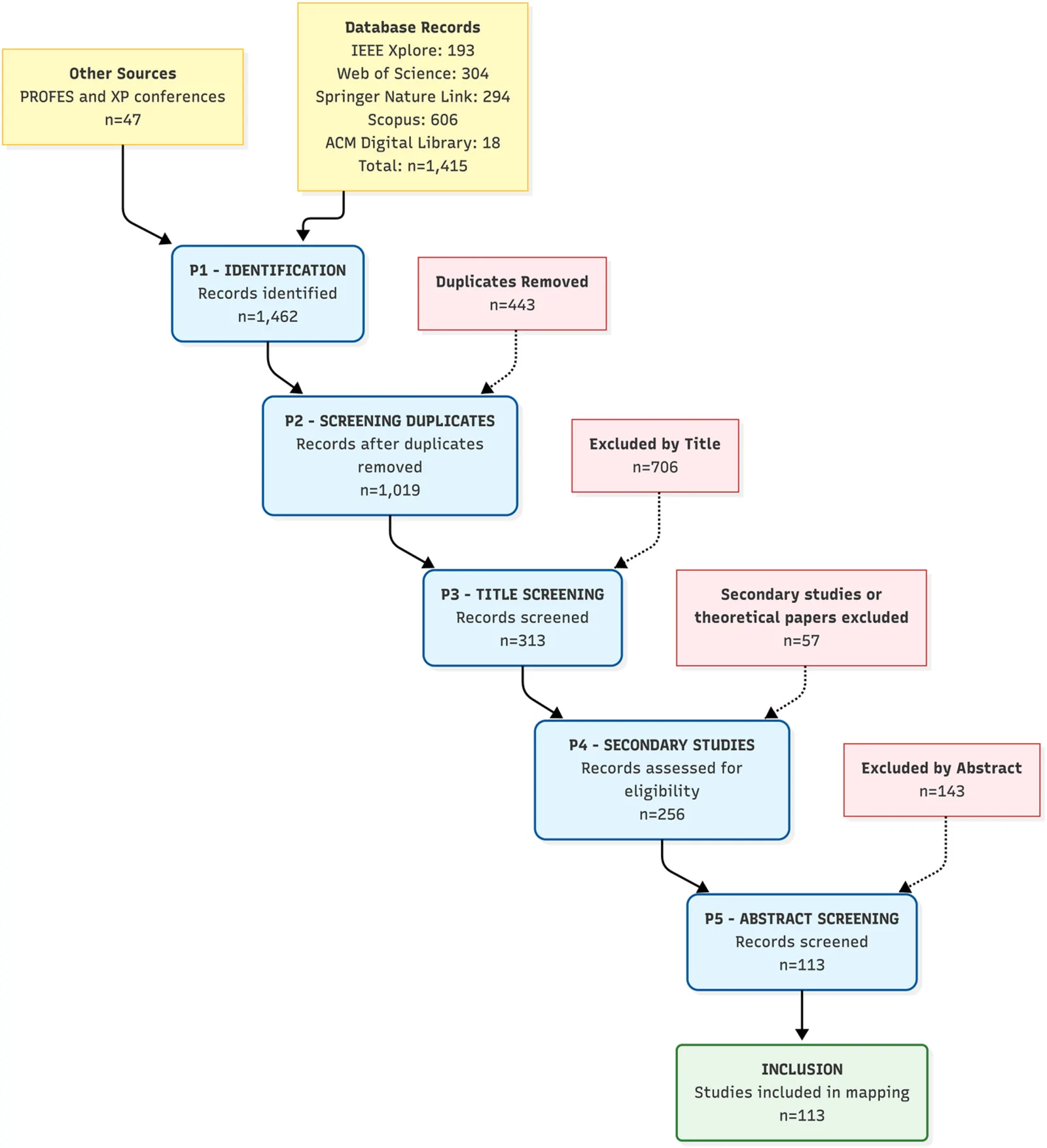
Screening flow diagram.

A Large Language Model, NotebookLM [9], was used as coding assistant to scan PDFs for metadata and thematic keywords. To ensure the integrity of the data extraction process and mitigate the risk of hallucinations, the notebook was fed with research questions, the Characterization Scheme and the list of 113 primary studies to provide a closed working context using specific source-anchoring. We employed context-bound prompts designed to require the model to cite specific passages from the provided sources for every data point extracted. Specifically, three strategies were employed:

• Context-bound prompts designed to require the notebook to use the specific sources and cite specific passages.
• An iterative prompt refinement phase was conducted on a control set of 10 primary studies to calibrate prompts against manual extraction. Prompts were iteratively refined until they achieve a target threshold of at least 80 % match agreement per data point across the 36 attributes of the control set, the minimum acceptable level commonly recommended for inter-rater agreement [10]. This threshold acted as a pass/fail quality gate: once it was met, the prompts were run against the complete corpus of 113 primary studies, followed by final human validation. The exact agreement value of the final iteration was not recorded separately.
• Manual validation was performed for all quantitative data points and qualitative classifications; while the AI proposed data points, the final decision for inclusion was made by a human researcher after a guided review of the source articles.

Additionally, the notebook’s responsibilities were limited to:

• Pattern recognition, scanning full-text PDFs for specific metadata and thematic keywords.
• Structured extraction, converting unstructured narrative findings and conclusions into structured records.
• Syntactic processing, summarizing qualitative descriptions into concise categorical statements.

This process was governed by a strict author-in-the-loop protocol.

## Limitations

A primary limitation of this dataset is the industry concentration within the source corpus; a significant portion of the primary studies originates from the financial sector or companies with long-standing engineering traditions, such as telecommunications and automotive. Consequently, the data may exhibit biases that do not fully reflect the dynamics of smaller startups or organizations without legacy infrastructure.

Furthermore, the dataset reveals a distinct quantitative gap, as most of the primary literature consists of qualitative experience reports and perception-based surveys rather than verifiable hard metrics or longitudinal quantitative data. This affects the availability of objective performance values for certain tailoring techniques.

The selection of primary studies was conducted through a single-reviewer design led by the first author, who served as the primary rater for all five screening phases. To ensure the objectivity and consistency of this process, we utilized a structured Characterization Scheme with predefined inclusion and exclusion criteria. We acknowledge that the absence of independent double-screening means that inter-rater reliability statistics cannot be reported. This represents a recognized methodological limitation that may introduce a degree of researcher bias into the corpus selection.

Additionally, despite the use of an extensive search string, the inherent ambiguity of terminology in the field introduces a potential risk that some relevant empirical studies may have been omitted during the collection phase. Finally, while a standardized Characterization Scheme was utilized, the thematic categorization of unstructured narrative text from the source papers involves an unavoidable degree of subjectivity inherent in qualitative data extraction.

## Ethics Statement

The authors have read and follow the ethical requirements for publication in Data in Brief. This work does not involve human subjects, animal experiments or any data collected from social media platforms.

## Declaration of Generative AI and AI-assisted Technologies in the Manuscript Preparation Process

During the preparation of this work, the authors used Claude (Anthropic) in order to proofread the manuscript for grammar, vocabulary, punctuation and terminology consistency. The tool did not generate scientific content, data, analyzes, interpretations or references. After using this tool, the authors reviewed and edited the content as needed and take full responsibility for the content of the published article.

## CRediT authorship contribution statement

**F. Gracia-Ahufinger:** Conceptualization, Formal analysis, Investigation, Data curation, Writing – original draft. **JAVIER J. Gutiérrez:** Conceptualization, Methodology, Validation, Writing – review & editing, Supervision. **J.A. García-García:** Conceptualization, Methodology, Validation, Writing – review & editing, Supervision. **N. Sánchez-Gómez:** Methodology, Validation, Writing – review & editing. **M.J. Escalona-Cuaresma:** Validation, Writing – review & editing.
